# Bio-fabrication of gold nanoparticles using aqueous extract of red tomato and its use as a colorimetric sensor

**DOI:** 10.1186/1556-276X-8-181

**Published:** 2013-04-19

**Authors:** Gadadhar Barman, Swarnali Maiti, Jayasree Konar Laha

**Affiliations:** 1 Department of Chemistry, Midnapore College, Midnapore, West Bengal 721101, India

**Keywords:** Gold nanoparticles, Green synthesis, Sodium dodecyl sulfate, Colorimetric sensor, Detection of pesticide, Estimation of pesticide, Calibration curve

## Abstract

In this work, we report a green method for the synthesis of gold nanoparticles (GNP) using the aqueous extract of red tomato (*Lycopersicon esculentum)*. We believe that citric acid and ascorbic acid present in tomato juice are responsible for the reduction of gold ions. This biosynthesized GNP in the presence of sodium dodecyl sulfate has been used as a colorimetric sensor to detect and estimate the pesticide, methyl parathion. The GNP in the presence of methyl parathion shows a new peak at 400 nm due to the formation of 4-nitrophenolate ion by catalytic hydrolysis of methyl parathion in alkaline medium. A calibration curve between the absorption coefficients of the 400-nm peak versus the concentration of the pesticide allows the quantitative estimation of the 4-nitrophenolate ion, thereby enabling indirect estimation of methyl parathion present in the system.

## Background

The last 2 decades have witnessed rapid advancement in various technologies for the fabrication of nanoparticles. Among the various classes of nanoparticles, metal nanoparticles are receiving much attention due to their application in various fields of science and technology. A number of approaches are available for the synthesis of silver and gold nanoparticles, for example, reduction of solution [1-3]; thermal [4], electrochemical [5], and sonochemical decomposition [6]; microwave-assisted synthesis [7]; and recently, using of green chemistry [8-11]. Using plants in the biosynthesis of metal nanoparticles, especially gold and silver nanoparticles, has received more attention as suitable alternative to chemical procedures and physical methods. Bioreduction of metal nanoparticles using a combination of biomolecules found in plant extract, e.g., enzymes, proteins, amino acids, vitamins, polysaccharides, and organic acids such as citrates is environmentally benign yet chemically complex. Extracts from plants may act as both reducing and capping agents in nanoparticle synthesis. Gardea-Torresdey et al. [12,13] were the first to report the formation of gold and silver nanoparticles inside living plants. A rapid reduction of the silver ions was observed when the silver nitrate solution comes to contact with geranium leaf extract [14]. A competition reduction of Au^3+^ and Ag^+^ ions was observed when presented simultaneously in neem (*Azadirachta indica)* leaf extract [15]. A simple biosynthesis procedure of applying green tea extract has been used for gold and silver nanoparticle synthesis by Vilchis-Nestor et al. [16].

In this work, we report a green method for the synthesis of gold nanoparticles (GNP) using the aqueous extract of red tomato (*Lycopersicon esculentum)*. The tomato is a member of the *Solanaceae* family. Nutritionally, the tomato is a good source of vitamins A and C [17]. Composition data vary due to the wide range of species, stage of ripeness, year of growth, climatic conditions, light, temperature, soil, fertilization, irrigation, and other conditions of cultivation, handling, and storage [18]. Average dry matter content of the ripe fresh food is between 5.0% and 7.5% [19].

The pectins, arabinogalactans, xylans, arabinoxylans, and cellulose are the major polysaccharides present in tomato. Glutamic acid comprises up to 45% of the total weight of free amino acids in fresh tomato juice with the next highest in concentration being aspartic acid. Citric acid is the most abundant organic acid with some malic acid also present [17]. Thus, the water extract of the tomato juice mostly contains proteins and water-soluble organic acids like citric acid, malic acid, amino acids, and vitamins. We believe that the presence of citric acid and ascorbic acid in the aqueous extract of tomato juice is responsible for the reduction of gold ions while the soluble proteins and amino acids are responsible for the stabilization of GNP.

This biosynthesized GNP in the presence of sodium dodecyl sulfate (SDS) has been used as a colorimetric sensor for the detection and estimation of the pesticide present in water and in alkaline medium. The pesticide methyl parathion is chosen because it is a highly neurotoxic agricultural chemical that is used extensively worldwide to control a wide range of insect pests. Its residue in the soil causes pollution in the environment and poses a serious risk to human health. The sensor properties were studied by examining the UV-visible spectral change due to the addition of methyl parathion at parts per million (ppm) levels.

## Methods

Chloroauric acid and SDS, both of AR grade, were purchased from Sigma-Aldrich Chemical Ltd. (Powai, Mumbai, India). Sodium hydroxide and methyl parathion were purchased from Merck (Whitehouse Station, NJ, USA). Double-distilled deionized water was used in all experiments.

The red tomato (*Lycopersicon esculentum*) was collected from the local market and washed with double-distilled deionized water. The skin was removed from the tomato, and the whole mass was squeezed to get the juice. This was filtered using a Whatman filter paper (Maidstone, UK) and diluted with twice its volume of water. This solution was used as the tomato extract. From this extract, we prepared diluted extract having different compositions like 5:5 (5 ml extract and 5 ml water), 6:4 (6 ml extract and 4 ml water), 7:3 (7 ml extract and 3 ml water), 8:2 (8 ml extract and 2 ml water), 10:0 (10 ml extract ), and so on.

**Figure 1:**
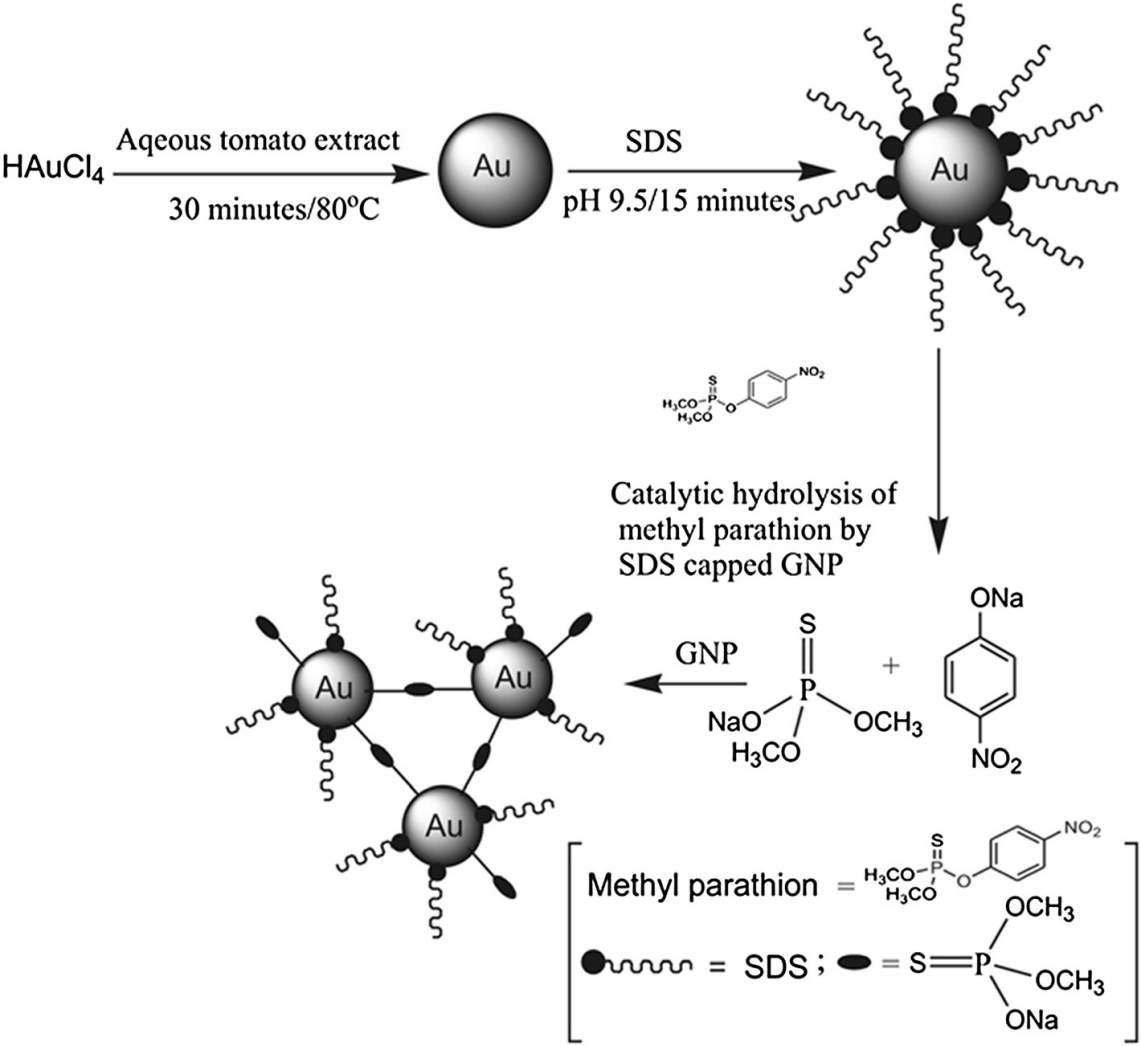
Schematic diagram of formation of GNP, catalytic hydrolysis of methyl parathion and aggregation of GNP.

GNP was produced by the reduction of chloroauric acid solution using this extract (Figure 1). Ten milliliters of the extract was cooled in ice-cold water, and 5 ml of a 3×10^-3^ (M) aqueous chloroauric acid was added dropwise with continuous stirring. The mixture was then cooled further for 10 min, and finally, it was heated for 30 min at 80°C. The color of the solution gradually changed from yellow to deep reddish violet. The reddish violet color indicated the formation of GNP.

The absorbance spectra of the GNP were analyzed using a Shimadzu UV-1800 spectrophotometer (Chestnut Ridge, NY, USA), and transmission electron microscopy (TEM) images were taken using a JEOL JEM-2100 high-resolution transmission electron microscope (HR-TEM, Akishima-shi,Japan). Samples for the TEM studies were prepared by placing a drop of the aqueous suspension of GNP on a carbon-coated copper grid followed by solvent evaporation under a vacuum. The crystalline nature of the GNP was examined using an X’Pert Pro X-ray diffractometer operated at a voltage of 40 kV and a current of 30 mA with CuK_α_ radiation.

3Ten milliliters of the as-prepared GNP was added to an equal volume of 3×10^-3^ (M) concentration of alkaline SDS. The pH of the solution was maintained at 9 to 9.5 by varying the amount of NaOH solution (0.15 (M)) added. The mixture was heated at 80°C for 30 min during which the color of the mixture deepened. This solution was used to detect the presence of methyl parathion. The concentration of methyl parathion in the alkaline GNP solution was varied from 0 to 200 ppm. Five hundred microliters of a solution containing different concentrations of methyl parathion was added to 5 ml of alkaline GNP solution, and the mixture was heated for 5 min with stirring. The deep reddish-violet color changed into brownish red. The intensity of the brownish red gradually increased with the increase of methyl parathion.

## Results and discussion

Synthesis of nanoparticles is an important activity in modern nanotechnology, and the biosynthesis of nanoparticles using plant extracts is presently getting much attention. The development of biological processes for the synthesis of nanoparticles is evolving as an important branch of nanotechnology. The present study deals with the synthesis of gold nanoparticles (GNP) using aqueous tomato extract.

**Figure 2:**
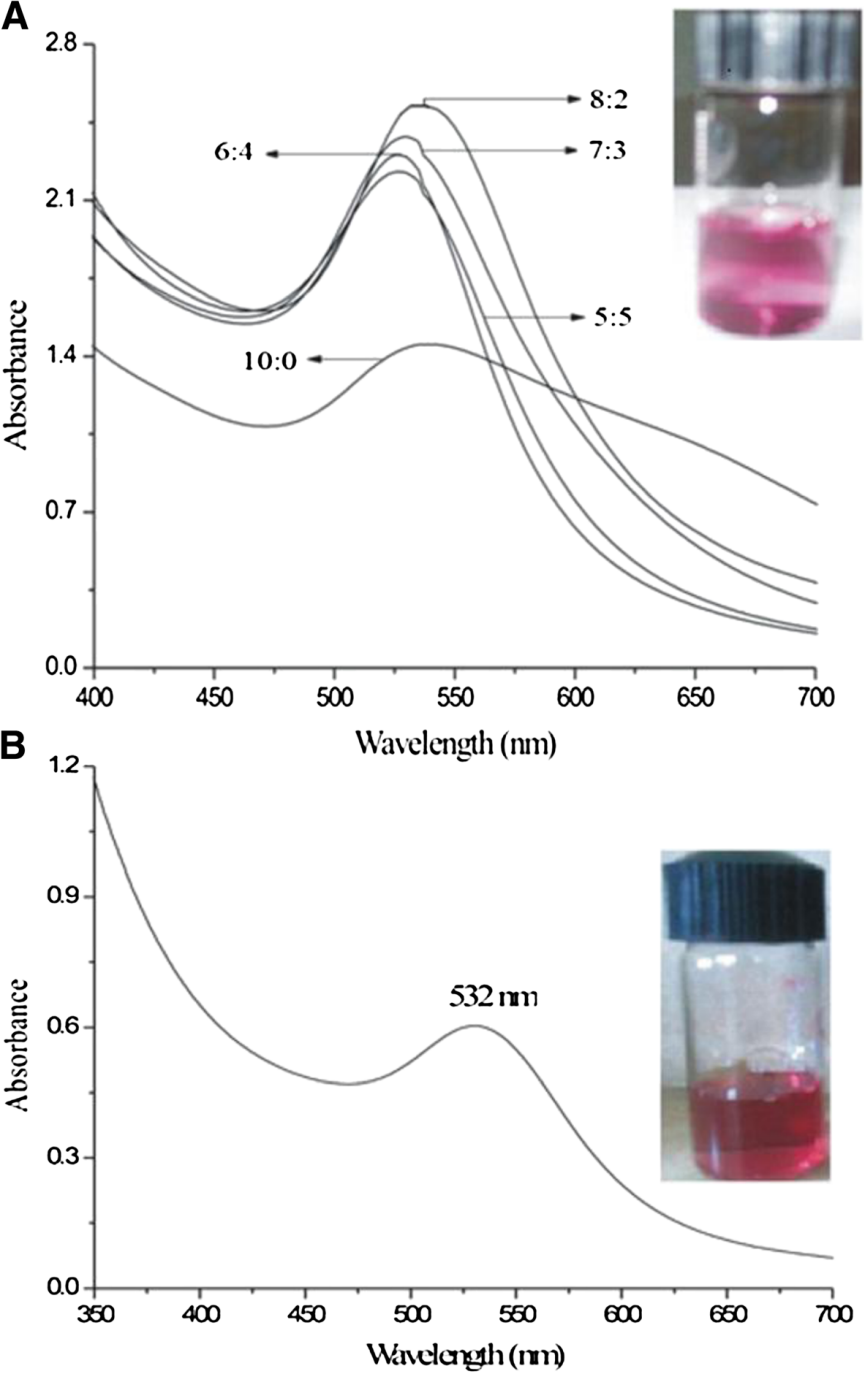
**UV–VIS absorption spectra of GNP at different compositions of tomato extract and SDS capped GNP in alkaline medium. **UV-VIS spectra of (**A**) GNP at different compositions and (**B**) SDS-capped GNP. Insets are digital photographic images of A and B.

The GNP produced exhibits reddish-violet color in water. The color appears due to the excitation of the localized surface plasmon vibrations of the metal nanoparticles (Figure 2A). A smooth and narrow absorption band was observed at 527 nm for the 5:5 extract composition and reaches 537 nm for the 8:2 composition. The plasmon band shifts to higher values with the increase of tomato concentration in the aqueous extract. At concentrations higher than this, the plasmon band shifts to 540 nm, and the extinction coefficient of the band decreases appreciably. Here, the tomato extract of 5:5 composition has been used throughout.

Shifting of gold plasmon band to the higher value may be explained as follows: tomato extract is a strong reducing agent but not a good capping agent. So, it induces rapid nucleation but cannot restrict the growth of gold nanoparticles. Hence, polydispersed gold nanoparticles are observed. When we use tomato extract (100%), the band shifts to 540 nm and the extinction coefficient decreases appreciably. This might be due to colloidal instability. The polydispersity and the colloidal instability (agglomeration tendency of gold nanoparticle) may be the reason for a broad spectrum of gold sol along with a shift in the peak position. The shifting of the peak position may be related to the increase of the size of gold nanoparticles.

**Figure 3:**
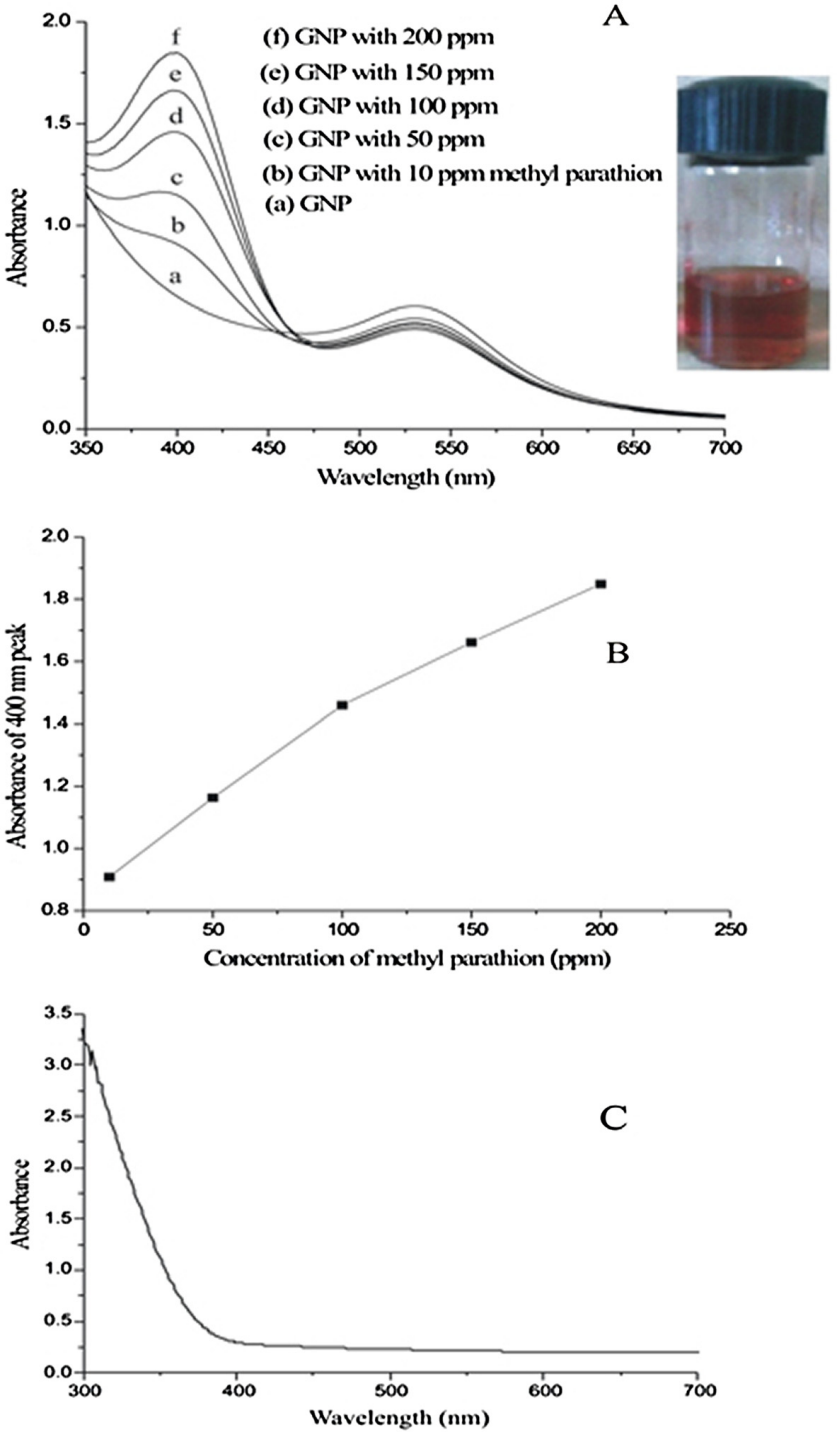
**UV–vis spectra of GNP and with methyl parathion, calibration curve (absorbance versus methyl parathion), and control spectrum. **(**A**) UV–vis spectra of GNP and GNP with various concentrations of methyl parathion 10 to 200 ppm; (inset) digital photographic images of color changes due to addition of methyl parathion. (**B**) Calibration curve between absorbance of 400-nm peak versus concentration of methyl parathion. (**C**) Spectrum of pure methyl parathion (control experiment).

To examine the sensor properties of the GNP, the solution was made alkaline by adding different amounts of NaOH (0.15 (M)). For these studies, the pH of the solution was maintained near 9 to 9.5 by adjusting the amount of NaOH in the solution, and a surfactant SDS was added to stabilize the medium. Here, SDS acts as a capping agent, due to which the SPR band shifts to 532 nm (Figure 2B). A comparatively sharp spectrum with absorbance at 532 nm was observed in this case. This can be explained from the fact that SDS, being a strong capping agent, stabilizes the gold nanoparticles as soon as nucleation happens and so restricts the maximum size of the nanoparticles. As a result, we obtained nearly monodispersed GNP. Methyl parathion was added to these alkaline solutions containing SDS in varying concentrations ranging from 10 to 200 ppm, and the change of absorption coefficient was observed. As soon as methyl parathion was added, we observed a new peak at around 400 nm in addition to the peak found at 532 nm. More interestingly, absorbance at 400 nm, the newly found peak, is seen to increase when the concentration of methyl parathion increased from 10 to 200 ppm (Figure 3A).

The newly found peak might be due to the 4-nitrophenolate ions which are produced by the hydrolysis of methyl parathion in alkaline medium [20]. It is known that due to the catalytic hydrolysis of methyl parathion, two hydrolyzed products, 4-nitrophenolate ions and sodium di-O-methyl thiophosphonate are produced (Figure 1). The literature confirms that 4-nitrophenolate ion shows a characteristic absorption peak at 400 nm [21]. The increase in the concentration of methyl parathion in the mixture quantitatively increases the amount of the 4-nitrophenolate ions in the medium which are reflected in the absorption spectra (Figure 3A).

A calibration curve between the absorption coefficient of the 400-nm peak and the concentration of the pesticide allows quantitative estimation of the methyl parathion present in a sample at ppm levels (Figure 3B). This calibration curve enables the estimation of methyl parathion indirectly by estimating the 4-nitrophenolate ions present in the medium. The corresponding decrease in the absorption peak of GNP at 532 nm may be due to the agglomeration of GNP which is facilitated by the presence of the other hydrolyzed product sodium di-O-methyl thiophosphonate containing sulfur. The formation of agglomeration of GNP is indicated by the broadening of the 532-nm peak in the presence of methyl parathion. A control experiment (Figure 3C) was carried out by taking methyl parathion only, and no peak was found at 400 nm. The peak at 400 nm emerges only when the hydrolysis of methyl parathion occurs in the presence of GNP in water.

**Figure 4:**
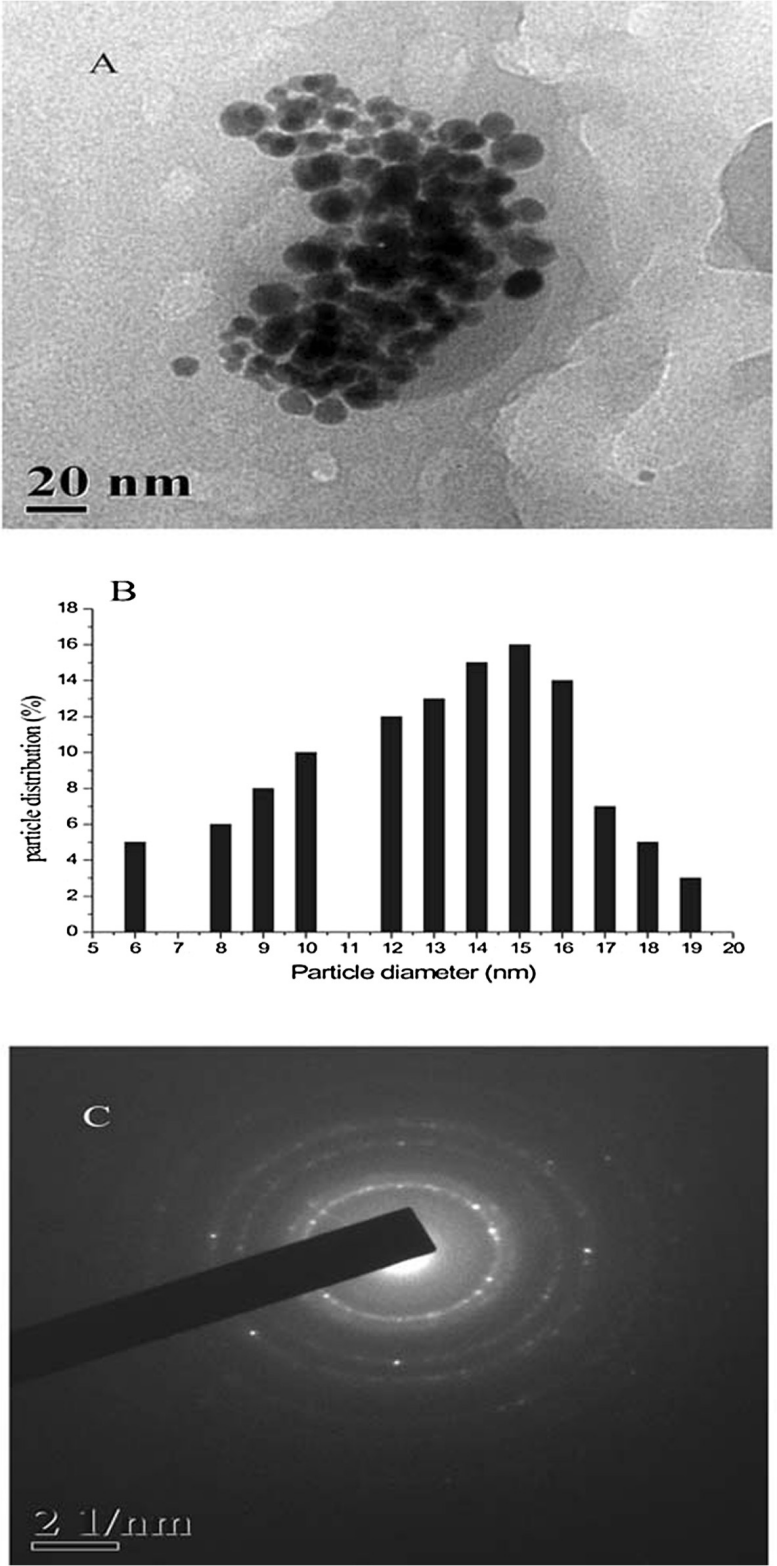
**TEM micrographs, particle size distribution histogram, and SAED pattern of GNP. **(**A**) TEM micrographs of GNP with tomato extract. (**B**) Particle size distribution histogram of spherical GNP, and (**C**) corresponding SAED pattern of GNP.

Figure 4A shows the TEM images of GNP produced from a 5:5 composition of tomato extract. The particles are mostly spherical, and their sizes varied from 5 to 20 nm. A histogram plot (Figure 4B) shows the distribution of particles of different sizes. Selected area diffraction (SAED) pattern shown in Figure 4C illustrates the crystalline nature of GNP.

**Figure 5:**
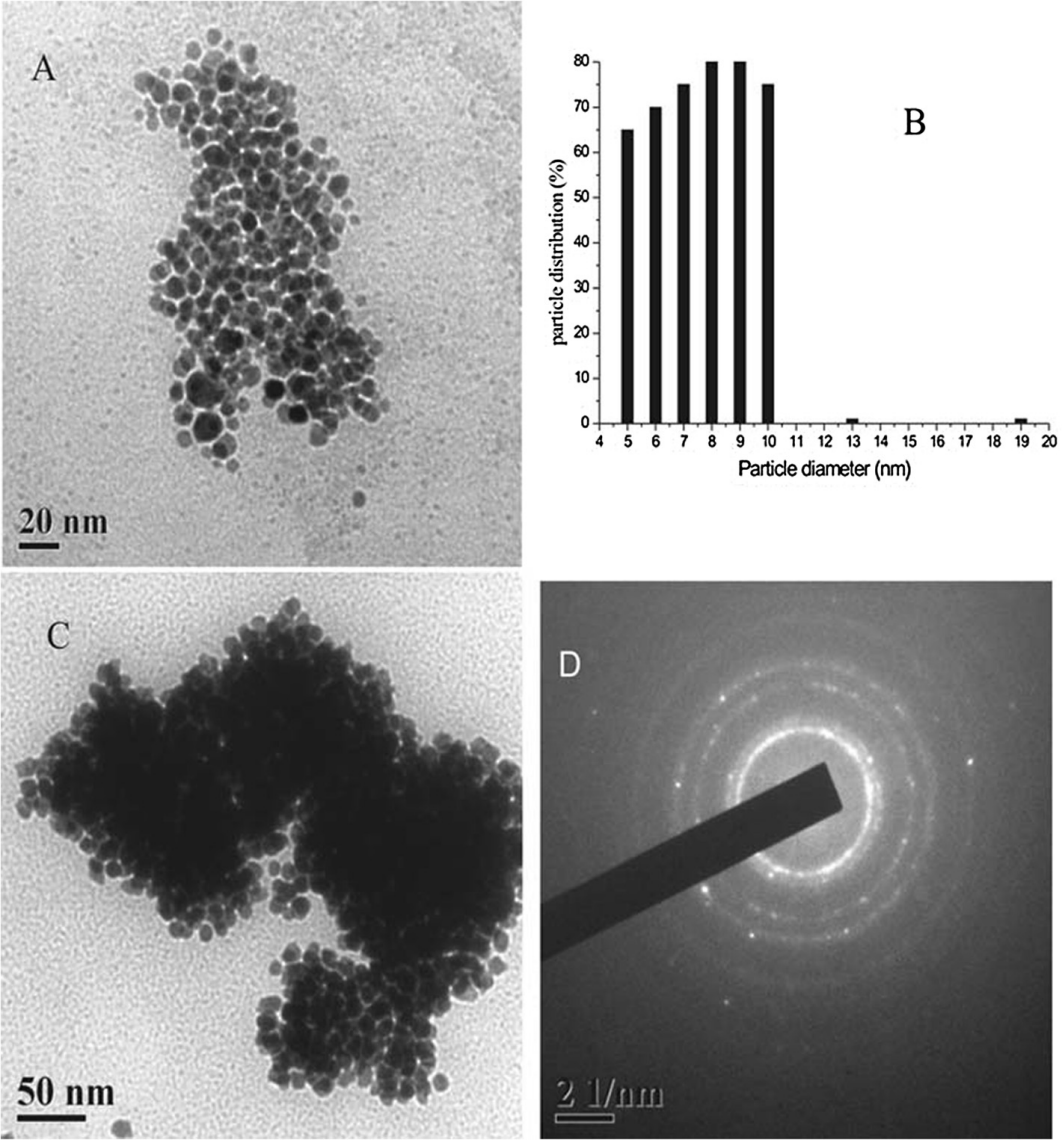
**TEM micrographs, particle size distribution histogram, SDS-capped GNP with methyl parathion, and SAED pattern of GNP. **(**A**) TEM micrographs of SDS-capped GNP with tomato extract. (**B**) Particle size distribution histogram of spherical GNP. (**C**) SDS-capped GNP in the presence of methyl parathion, and (**D**) corresponding SAED pattern of GNP.

Figure 5A shows the representative TEM images of GNP with SDS in alkaline medium. The histogram of it is shown in Figure 5B. The significant changes are observed in the size of the particles. The particles become of uniform sizes, and the sizes reduced to 5 to 10 nm. SDS, being a strong capping agent stabilizes the gold nanoparticles as soon as nucleation happens and thereby restricts the nanoparticles to a finite size. As a result, nearly monodispersed gold nanoparticles of sizes 5 to 10 nm were obtained.

The TEM image of Figure 5C is due to GNP with methyl parathion in alkaline medium in the presence of SDS. It appears that the restructuring of GNP occurs after the addition of methyl parathion and agglomeration of particles is observed. It is likely that the surface of the GNP forms an Au-S coordination bond as the sol is being heated after addition of methyl parathion and some hydrolyzed product sodium di-O-methyl thiophosphonate get adsorbed on the Au surface by replacing SDS. As it is anionic in alkaline medium, its adsorption on the GNP surface lowers the surface charge, and thus, they agglomerate and particle clustering is observed (Figure 1).

**Figure 6:**
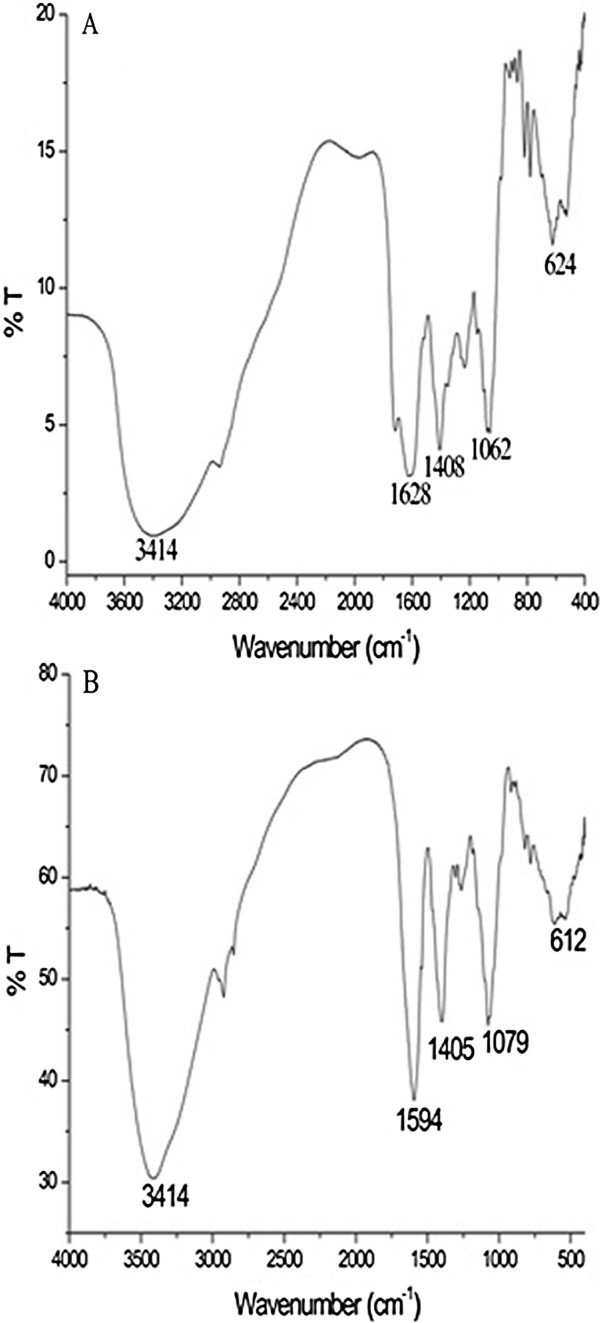
**FTIR spectra of vacuum-dried powder of red tomato and GNP synthesized from aqueous red tomato extract. **(**A**) FTIR spectra of vacuum-dried powder of red tomato (*Lycopersicon esculentum) *and (**B**) GNP synthesized from aqueous red tomato extract.

Fourier transform infrared spectroscopy (FTIR) analysis was performed to identify the biomolecules localized on the surface and responsible for the reduction of gold solution. Representative FTIR spectra of pure tomato extract and the as-prepared GNP are shown in Figure 6A,B, respectively. The spectrum of the dried aqueous extract of tomato juice shows a number of frequencies in the range 1,800 to 1,000 cm^-1^ corresponding to C=O stretching (1,720 cm^-1^) of organic acid present, secondary ammine (1,628 cm^-1^) from the proteins present in the extract. In comparison with the spectra, it is evident that the peak (1,720 cm^-1^) due to the acid groups present in tomato extract is missing in the GNP spectrum which conforms that these groups are responsible for reduction. The shifting of bands from 1,628 to 1,594 cm^-1^, 1,408 to 1,405 cm^-1^, and 1,062 to 1,079 cm^-1^ indicates the direct involvement of proteins in stabilizing the sol particles [22].

**Figure 7:**
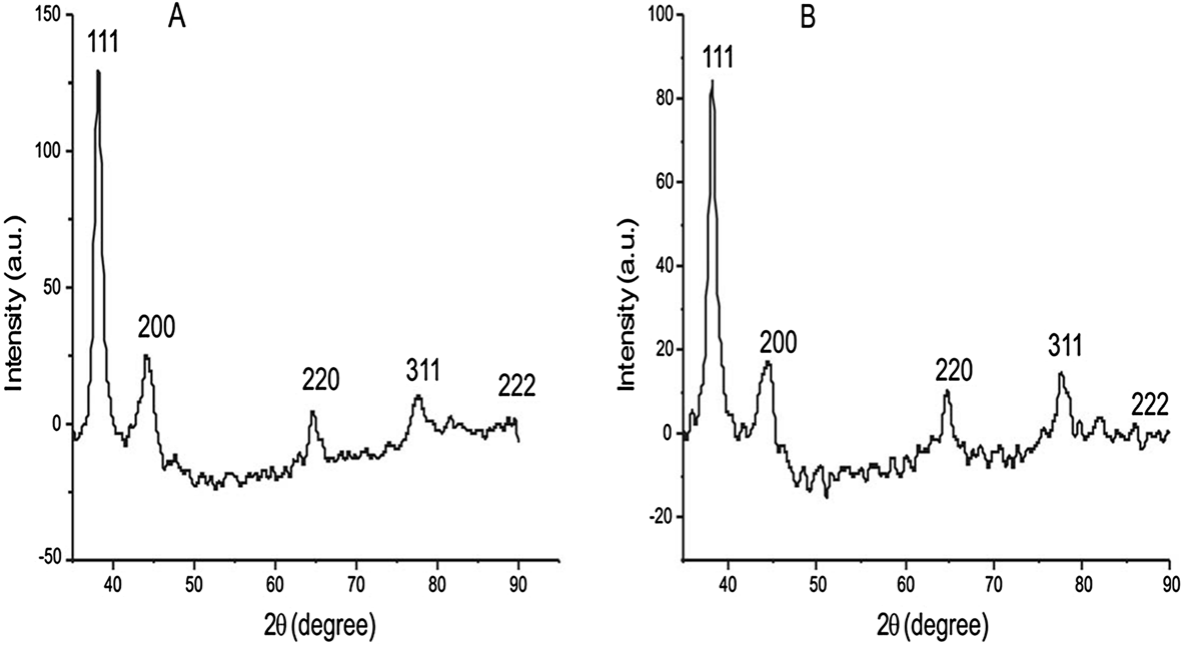
**XRD of SDS capped GNP and GNP in presence of methyl parathion. **XRD of GNP (**A**) before and (**B**) after addition of methyl parathion.

The XRD analysis was performed to confirm the crystalline nature of biologically synthesized GNP. Various Bragg reflections are clearly visible in the gold XRD pattern (Figure 7A) which indicates the face-centered cubic (FCC) structure of the bulk gold having peaks at 38.21°, 44.29°, 64.68°, and 77.61° corresponding to (111), (200), (220), and (311) planes, respectively. The XRD spectrum of the GNP after reaction with methyl parathion is shown in Figure 7B, and it is visible that the spectrum shows the same four peaks. On the basis of these Bragg reflections, we can say that biologically synthesized GNP have FCC structures, essentially crystalline in nature, and are mostly (111)-oriented.

## Conclusions

A green method has been used for the synthesis of gold nanoparticles using the aqueous extract of red tomato. Citric acid and ascorbic acid present in tomato juice are believed to be responsible for the reduction of gold ions. This biosynthesized GNP has been used as colorimetric sensor for detection and estimation of methyl parathion present in water in the presence of SDS. A new peak generated at 400 nm due to the formation of 4-nitrophenolate ion when methyl parathion added in the alkaline medium of the GNP. The variations of the absorbance of this peak have been used for estimation of methyl parathion present in the solution. To quantitatively estimate methyl parathion present in water, a calibration curve between the absorbance of 400-nm peak versus concentration of methyl parathion has been drawn.

## Competing interests

The authors declare that they have no competing interests.

## Authors’ contributions

GB carried out the experiment. GB and SM drafted the manuscript. JKL guided the research and modified the manuscript. All authors read and approved the final manuscript.

## Authors’ information

JKL is Associate Professor and Head of the Department of Chemistry, Midnapore College, West Bengal, India. GB and SM are research scholars of this department.

## References

[B1] Pal T Sau TK Jana NR Reversible formation and dissolution of silver nanoparticles in aqueous surfactant media Langmuir 1997 8 1481 1485 10.1021/la960834o

[B2] Goia DV Matijevic E Formation mechanisms of uniform colloid particles New J Chem 1998 8 1203 1215 10.1039/a709236i

[B3] Munro CH Smith WE Garner M Clarkson J White PC Characterization of the surface of a citrate-reduced colloid optimized for use as a substrate for surface-enhanced resonance Raman scatterings Langmuir 2002 8 3712 3720

[B4] Esumi K Tano T Torigoe K Meguro K Preparation and characterization of bimetallic palladium-copper colloids by thermal decomposition of their acetate compounds in organic solvents Chem mater 1990 8 564 587 10.1021/cm00011a019

[B5] Rodriguez-Sanchez ML Blanco MC Lopez-Quintela MA Electrochemical synthesis of silver nanoparticles J Phys Chem B 2000 8 9683 9688 10.1021/jp001761r

[B6] Zhu J Liu S Palchik O Koltypin Y Gedanken A Shape-controlled synthesis of silver nanoparticles by pulse sonoelectrochemical methods Langmuir 2000 8 6396 6399 10.1021/la991507u

[B7] Pastoriza-Santos I Liz-Marzan LM Formation of PVP-Protected Metal Nanoparticles in DMF Langmuir 2002 8 2888 2894 10.1021/la015578g

[B8] Chandran SP Chaudhary M Pasricha R Ahmad A Sastry M Synthesis of gold nanotriangles and silver nanoparticles using aloe vera plant extract Biotechnol Prog 2006 8 577 583 10.1021/bp0501423 16599579

[B9] Shiv Shankar S Rai A Ahmad A Sastry M Controlling the optical properties of lemongrass extract synthesized gold nanotriangles and potential application in infrared-absorbing optical coatings Chem Mater 2005 8 566 572 10.1021/cm048292g

[B10] Rai A Singh A Ahmad A Sastry M Role of halide ions and temperature on the morphology of biologically synthesized gold nanotriangles Langmuir 2006 8 736 741 10.1021/la052055q 16401125

[B11] Ankamwar B Damle C Ahmad A Sastry M Biosynthesis of gold and silver nanoparticles using Emblica Officinalis fruit extract, their phase transfer and transmetallation in an organic solution J Nanosci Nanotechnol 2005 8 1665 1671 10.1166/jnn.2005.184 16245525

[B12] Gardea-Torresdey JL Parsons JG Gomez E Peralta-Videa JR Troiani H Santiago P Jos’e-Yacam’an M Formation and growth of Au nanoparticles inside live Alfalfa plants Nano Lett 2002 8 397 401 10.1021/nl015673+

[B13] Gardea-Torresdey JL Gomez E Peralta-Videa JR Parsons JG Troiani H Santiago P Jos’e-Yacam’an M Alfalfa sprouts: A natural source for the synthesis of silver nanoparticles Langmuir 2003 8 1357 1361 10.1021/la020835i

[B14] Shiv Shankar S Ahmad A Sastry M Geranium leaf assisted biosynthesis of silver nanoparticles Biotechnol Prog 2003 8 1627 1631 10.1021/bp034070w 14656132

[B15] Shiv Shankar S Rai A Ahmad A Sastry M Rapid synthesis of Au, Ag and bimetallic Au core Ag shell nanoparticles using Neem (Azadirachta indica) leaf broth J Colloid Interf Sci 2004 8 496 502 10.1016/j.jcis.2004.03.003 15178278

[B16] Vilchis-Nestor AR Sanchez-Mendieta V Camacho-Lopez MA Gomez-Espinosa RM Camacho-Lopez MA Arenas-Altorre JA Solventless synthesis and optical properties of Au and Ag nanoparticles using Camellia sinensis extract Mater Lett 2008 8 3103 3105 10.1016/j.matlet.2008.01.138

[B17] Gould WA Tomato Production, Processing and Quality Evaluation 1983 2 Westport, CT: AVI Publishing Company, Inc 3 50

[B18] Yilmaz E The chemistry of fresh tomato flavor Turk J Agric For 2001 8 149 155

[B19] Petro-Turza M Flavor of tomato and tomato products Food Rev Int 1986 8 311 353

[B20] Scott AT Rafaela N Martine M Dan Z Eddie C Andrew DK Accelerating the initial rate of hydrolysis of methyl parathion with laser excitation using monolayer protected 10 nm Au nanoparticles capped with Cu(bpy) catalyst Chem Comm 2012 8 4121 4123 10.1039/c2cc30850a 22434011

[B21] Chen C Chen DH Spontaneous synthesis of gold nanoparticles on gum arabic–modification iron oxide nanoparticles as a magnetically recoverable nanocatalyst Nanoscale Res Lett 2012 8 1 7 10.1186/1556-276X-7-1 22713480 PMC3432631

[B22] Bar H Bhui DKR Sahoo GP Sarkar P Pyne S Chattopadhyay D Misra A Synthesis of gold nanoparticles of variable morphologies using aqueous leaf extracts of Cocculus hirsutus J Exp Nanosci 2012 8 109 119 10.1080/17458080.2010.509875

